# Quantifying PM_2.5_ capture capability of greening trees based on leaf factors analyzing

**DOI:** 10.1007/s11356-016-7687-9

**Published:** 2016-09-19

**Authors:** Dan Liang, Chao Ma, Yun-qi Wang, Yu-jie Wang, Zhao Chen-xi

**Affiliations:** 1 Soil and Water Conservation of Beijing Engineering Research Center, Beijing Forestry University, Beijing, 100083 China; 2 Chongqing Jinyun Forest Ecological Station, School of Soil and Water Conservation, Beijing Forestry University, Beijing, 100083 China; 3 Beijing Institute of Hydrogeology and Engineering Geology (Beijing Institute of Geo-Environment Monitoring), Beijing, China

**Keywords:** Beijing, Chongqing, PM_2.5_ capture capability, Deposition chamber, Leaf morphology, Groove proportion

## Abstract

As PM_2.5_ affect human health, it is important to target tree planting in the role of reducing air pollution concentrations. PM_2.5_ capture capability of greening trees is associated with leaf morphology, while quantitative research is scanty. In this paper, the PM_2.5_ capture capability of 25 species in Beijing and Chongqing were examined by a chamber device. Groove proportion, leaf hair, stomatal density, and stomata size were selected as indexes of leaf morphology. Results showed that groove proportion and stomata size significantly relate to PM_2.5_ capture quantity, while no significantly positive correlations were found for leaf hairs and stomatal density. Broadleaf species are conducive to PM_2.5_ capture for their rich leaf morphology and have an edge over coniferous in PM_2.5_ capture per leaf area. However, coniferous had a larger PM_2.5_ capture capability per tree due to the advantage of a large leaf area. Significant difference existed between the species in Beijing and Chongqing due to the different leaf morphology. Urban greening trees are diverse and the structures are complicated. Complex ecological environment may lead to different morphology characteristics. Climate and pollution conditions should be considered when greening.

## Introduction

Increasing PM_2.5_ concentrations have become the primary pollutants in many densely populated cities. In China, lots of cities are experiencing serious air pollution and bearing heavy burden of respiratory diseases (Shu et al. 2015; Fu et al. 2015). It has been documented that the incidence of lung cancer in Beijing was 1.055 for men and 1.149 for women due to 10 mg m^−3^ increase of PM_2.5_ concentration (Guo et al. 2014, 2016). Since 2001, the estimated average total mortality due to PM_2.5_ was approximately 5100 a year until 2012, and the unit capital mortality for all ages was around 15 in 10,000. Increasing PM_2.5_ concentration is the primary environmental problem, leading to an urgency of implementing air pollution abatement (Zheng et al. 2015).

Phytoremediation can clean air to a great extent depending on its capability of reducing speed velocity and capturing particles (Popek et al. 2015). The effectivity of trees to capture PM_2.5_ has been addressed a lot. Some authorities proposed tree planting as a dominant measure to alleviate airborne fine particulate matter. These plants commonly have a large surface area to filter PM out of the air by their removing or capturing capability derived from the leaf surface (Nowak et al. 2013; Chen et al. 2015). Understanding the PM_2.5_ capture capability of trees is crucial to assess the role of urban forest construction policies in reducing PM_2.5_ concentrations.

Many attempts have been made to quantify the effectiveness of urban trees in capturing PM_2.5_ (Dzierżanowski et al., 2011; Gromke and Ruck 2012; Sæbø et al. 2012; Speak et al. 2012; Popek et al. 2013). Although high effectiveness of trees has been demonstrated, significant differences between species were recorded. Both species and location to the pollution source are critical in determining the effectiveness (Pullman, 2009; Mori et al. 2015). Previous studies suggested that urban planting in the future should focus on the utility of conifers (Beckett et al. 1998; Beckett et al., 2000a, b). Species-specific features, such as leaf surface, leaf type, leaf area index, and leaf morphology, act as the main structures and are important factors affecting capture capability. Broadleaf species with rough leaf surfaces can capture more PM_2.5_ than those with smooth leaf surfaces (Nguyen et al. 2015), indicating that plant choices are important because proper or reasonably planned layout of them can maximize the efficiency in air pollution abatement (Fowler et al. 2004; Räsänen et al. 2013).

Pervious works qualitatively examined on the leaf roughness, leaf hair, stomatal density, and stomata size (Sæbø et al. 2012). However, a detailed quantitative research is necessary to analyze the influence of leaf morphology on PM capture capability. In this paper, the effectiveness of 25 tree species in capturing PM_2.5_ was examined using a chamber device. Tree leaves were sampled in growing season (e.g., from May to September), then dried and exposed to NaCl aerosol particles in the chamber device. Groove proportion, leaf hair, stomatal density, and stomata size were quantified using scanning electron microscopy (SEM). Capturing efficiencies were measured at controlled NaCl concentrations and duration and analyzed regarding leaf morphology.

## Materials and methods

### Study site and plant material

**Fig. 1:**
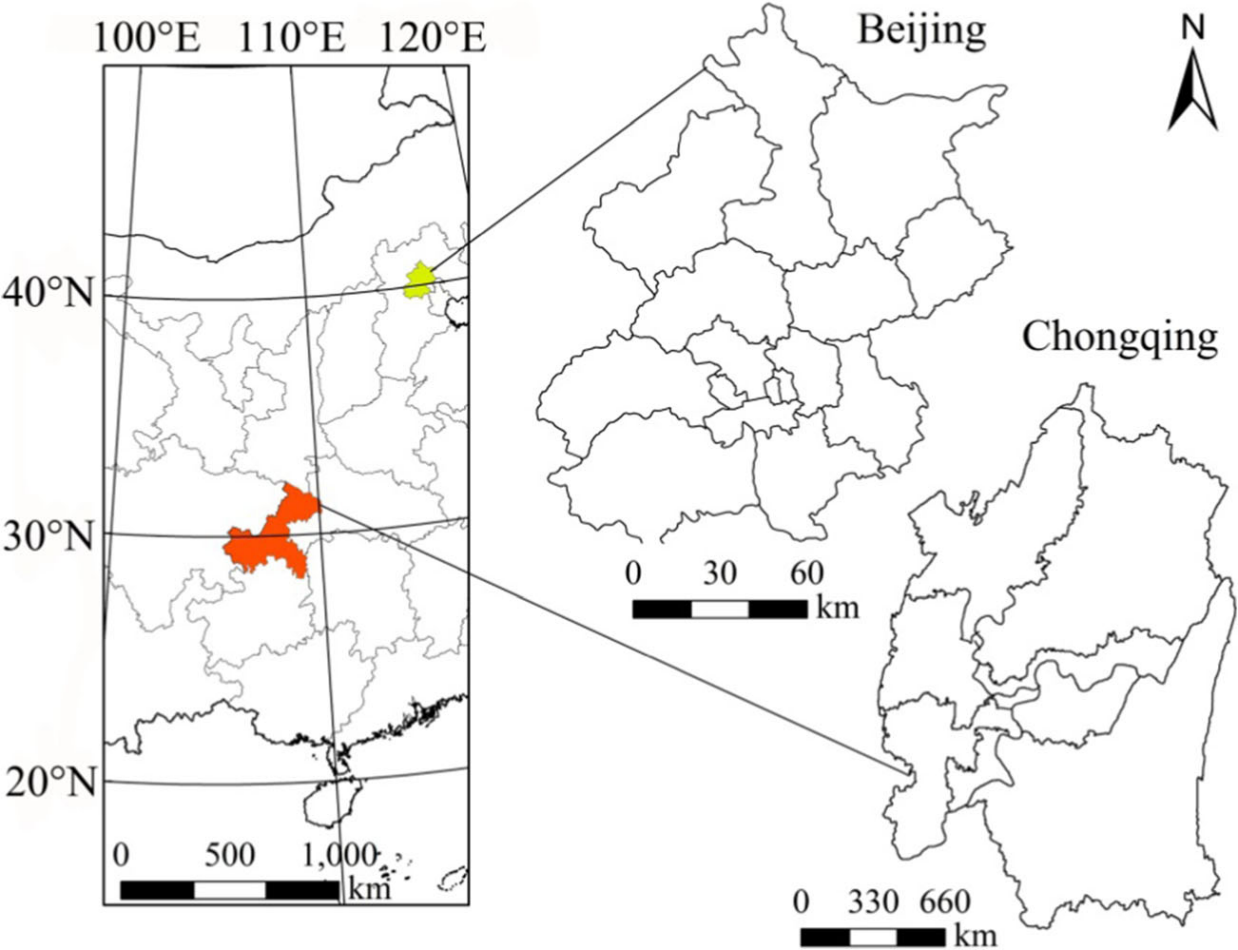
Location of the two sampling sites

Fifteen species in Chongqing and 10 in Beijing were tested (Table 2). Beijing is located in the northwest of China and is a densely populated city with monsoon climate and poor air quality (39.54° N, 116.23° E) (Fig. 1). Previous documents revealed that PM_2.5_ concentrations in Beijing have increased in recent years (the annual PM_2.5_ concentration in 2014 was 85.9 μg m^−3^, data from Environmental Protection Administration, China). Chongqing (29.59° E, 106.54° N) has a relatively clean and humid subtropical monsoon climate. Local annual PM_2.5_ concentration in Chongqing 2014 was 62.8 μg m^−3^ (data from Environmental Protection Administration, China). In addition, there are 5 common species among the total 25 species including *Platanus orientalis*, *Broussonetia papyrifera*, *Ginkgo biloba*, *Magnolia soulangeana*, and *Pinus massoniana*. Besides, 20 species are broadleaf species, others are conifer.

### Sampling

All trees distributed along urban main roads where heavy traffic pollution dominate. Daily meteorological data were obtained by an automatic weather station at a height of 1.5 m above the ground in both cities. Temperature, humidity, wind speed, and precipitation were recorded. Meteorological data and PM_2.5_ concentrations (Environmental Protection Agency, China) during May to September 2014 were shown in Table 1.

**Table 1:** Meteorological data and annual PM_2.5_ concentrations from May to September 2014

City	Growing season	Temperature (°C)	Humidity (%)	Wind speed (m s^−1^)	Precipitation (mm)	Average PM_2.5_ concentration (μg m^−3^)
Chongqing	May–September	25.2	77.8	1.3	713.3	60.5
Beijing	May–September	23.1	40.5	2.0	395.2	85.9

Four trees of each species were sampled twice a month from May to September. These trees were grown well, with similar age, and a diameter at breast height (*DBH*). Note that the first year twigs can provide better information on pollution than perennial twigs. We sampled the first year twigs from four directions at three heights (low, middle, and high layer of canopy), which were at a 1.0–2.0-m height above ground level depending on the tree structure. Twelve twigs in each tree were sampled with weight ranging from 300 to 500 g, then, washed with 500 mL deionized water in the laboratory. Lastly, 12 sampled twigs of each tree were dried in an incubator and exposed to PM_2.5_ in a chamber device.

We assumed that all leaves of a tree are comparable to sampled ones and exposed to the same pollution concentration in field conditions. In order to investigate the PM_2.5_ capture capability of a tree, we multiplied the masses of PM_2.5_ per unit leaf area by total leaf area. Leaf area index (*LAI*) and vertical projection area of the crown (*S*_*t*_) were applied to estimate total leaf area. A tape measure and DBH ruler were used in the field to measure the *DBH*, tree height (*H*), and crown diameter (*C*).

### Chamber device

**Fig. 2:**
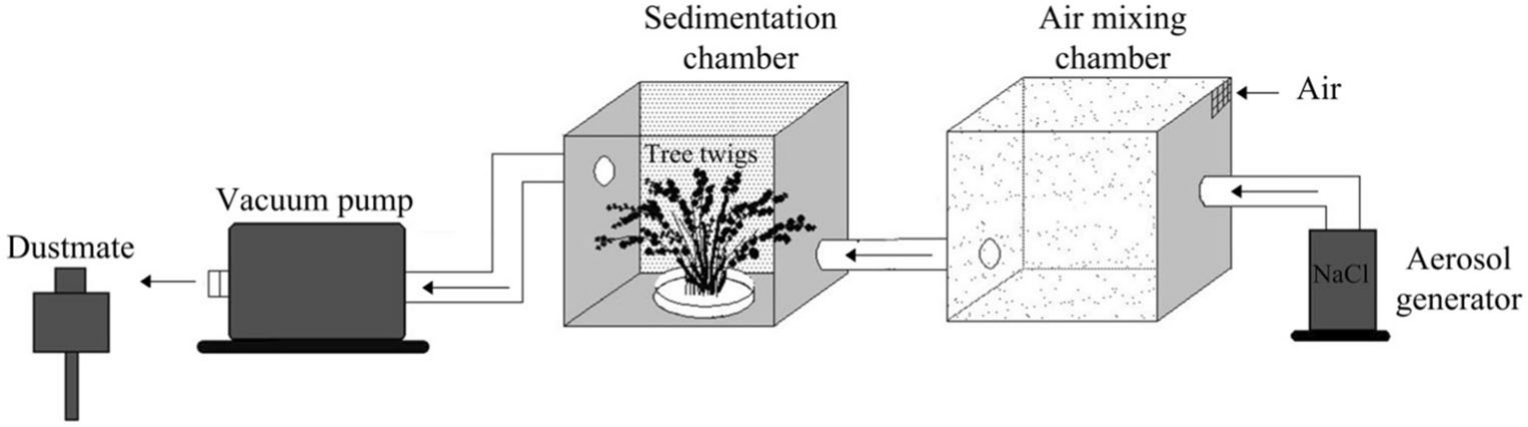
Components of chamber device (Vacuum pump, Tai guan JP-40V; Aerosol generator, SH600; sedimentation chamber and air mixing chamber, a cube of side length is 400 mm, which consistent with Hwang et al. (2011), made from acrylic board. Twigs were placed in the center of the sedimentation chamber)

Particles gradually accumulate on the surfaces of tree leaves until there is a dynamic equilibrium between deposition and loss (Mitchell et al. 2010). Aerosol particles with controlled concentration and size can be generated by an aerosol generator. In this research, sodium chloride (NaCl) solution with a concentration of 0.1 mol L^−1^ was chosen as the PM_2.5_ source (Freer-Smith et al. 2004) because the component of PM_2.5_ in Beijing and Chongqing mainly consists of sulfate, nitrate, black carbon, and organic pollutants (Guo et al. 2014), while Cl^−^ was less. Thus, there is no background interference (Beckett et al., 2000a, b; Freer-Smith et al. 2004). Pressure of aerosol generator was set to 25 psi, ensuring aerosol particle diameters were under 2.5 μm. PM_2.5_ concentration at downstream of the vacuum pump was measured 5 min at a time by DustMate (Turnkey Instruments, UK). Each test lasted about half an hour when PM_2.5_ concentrations remain stable. An average PM_2.5_ concentration of 550 μg m^−3^ was determined. After being exposed to PM_2.5_ in a sedimentation chamber (Fig. 2), twigs were washed by 500 mL deionized water and the filtrates were collected. Ion chromatograph (DionexICS-1600, USA) were used to detect the concentration of Cl^−^(mg mL^−1^).

### Statistics and classification of leaf morphology

Leaves were dried under air temperature of 80 °C in an oven. Two small square samples with a length of 5 mm and 1–2 cm to the central vein were cut from both leaf sides. Then, the samples were stick to the observation platform and plated with gold by ion-plating apparatus of Scan Electron Microscopy (SEM, HitachiS-3400 N, Japan). Afterward, we magnified them to 150–2000 times to observe the leaf morphology.

**Fig. 3:**
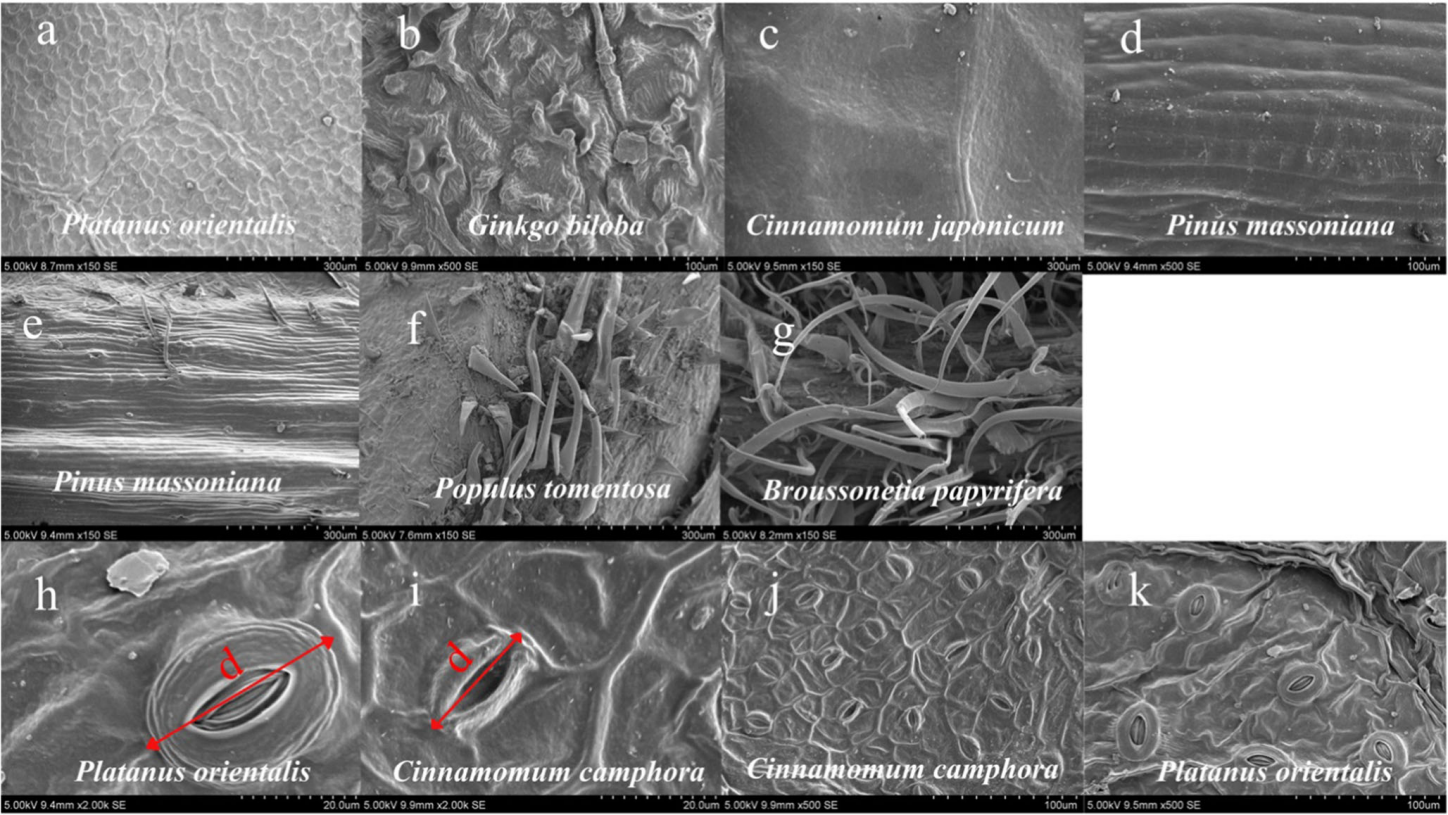
Typical leaf morphology of the tested trees (**a**–**d** represents net, nodular or verrucous, stripe and corrugated groove, respectively. **e**–**g** embodies the leaf hair difference which represents hairless, sparse hair and hairy, respectively. **h**–**k** showed different stomatal type. **h** and **i** represent the stomatal that major semi-axis greater than 20 μm and less than 20 μm, respectively. **j** and **k** refer to the stomatal density greater than 100 mm^−2^ and less than 100 mm^−2^, respectively)

Sampled 25 species have distinctive grooves (Fig. 3a–d), such as stripe, net, corrugated, nodular, or verruca, where PM_2.5_ is often deposited. Proportion of groove (*G*) is expressed as follows:

1 $$ G=\raisebox{1ex}{${A}_g$}\!\left/ \!\raisebox{-1ex}{${A}_t$}\right.\times 100\% $$

where *A*_*g*_ (μm^2^) represents the projection area of groove, *A*_*t*_ represents the area of the leaf sample (μm^2^).

Leaf hair per unit area (1 mm^2^) on both leaf sides was counted (Fig. 3e–g). It is noted that leaf hairs of most sampled trees are longer than 150 μm, while leaf hairs of some trees are shorter than 150 μm. A correction factor (c_1_ = 0.4) was introduced for the leaf hair shorter than 150 μm. The length of the major long axis was considered as the stoma size. Stoma number per unit leaf area was counted to calculate the stoma density (Fig. 3h–k).

### Data analysis

Leaves were dried by airing after washing. For broadleaf species, leaf area was obtained by the leaf area meter (YMJ-B). For needle leaves, an equation was used (Li et al. 2001). Captured PM_2.5_ per unit area (*APM*_*2.5*_) is expressed as:

2 $$ AP{M}_{2.5}=\raisebox{1ex}{${C}_iV$}\!\left/ \!\raisebox{-1ex}{${A}_i$}\right.\times {10}^6 $$

where *C*_*i*_ represents *Cl* ^−^ concentration in the filtrate and *V* represents the volume of the filtrate (500 mL). *A*_*i*_ represents the leaf areas. Total leaf area(*Y*) is represented as (Yang. 2011):

3 $$ Y={S}_t\cdot LAI $$

4 $$ {S}_t=\frac{1}{4}\pi {C}^2 $$

where *LAI* represents leaf area index, *S*_*t*_ represents vertical projection area of the crown, and *C* represents the average crown diameter. Then, captured PM_2.5_ per tree (*T*, mg) is calculated as follows:

5 $$ T=AP{M}_{2.5}\cdot Y $$

## Results

### Leaf area per tree

**Fig. 4:**
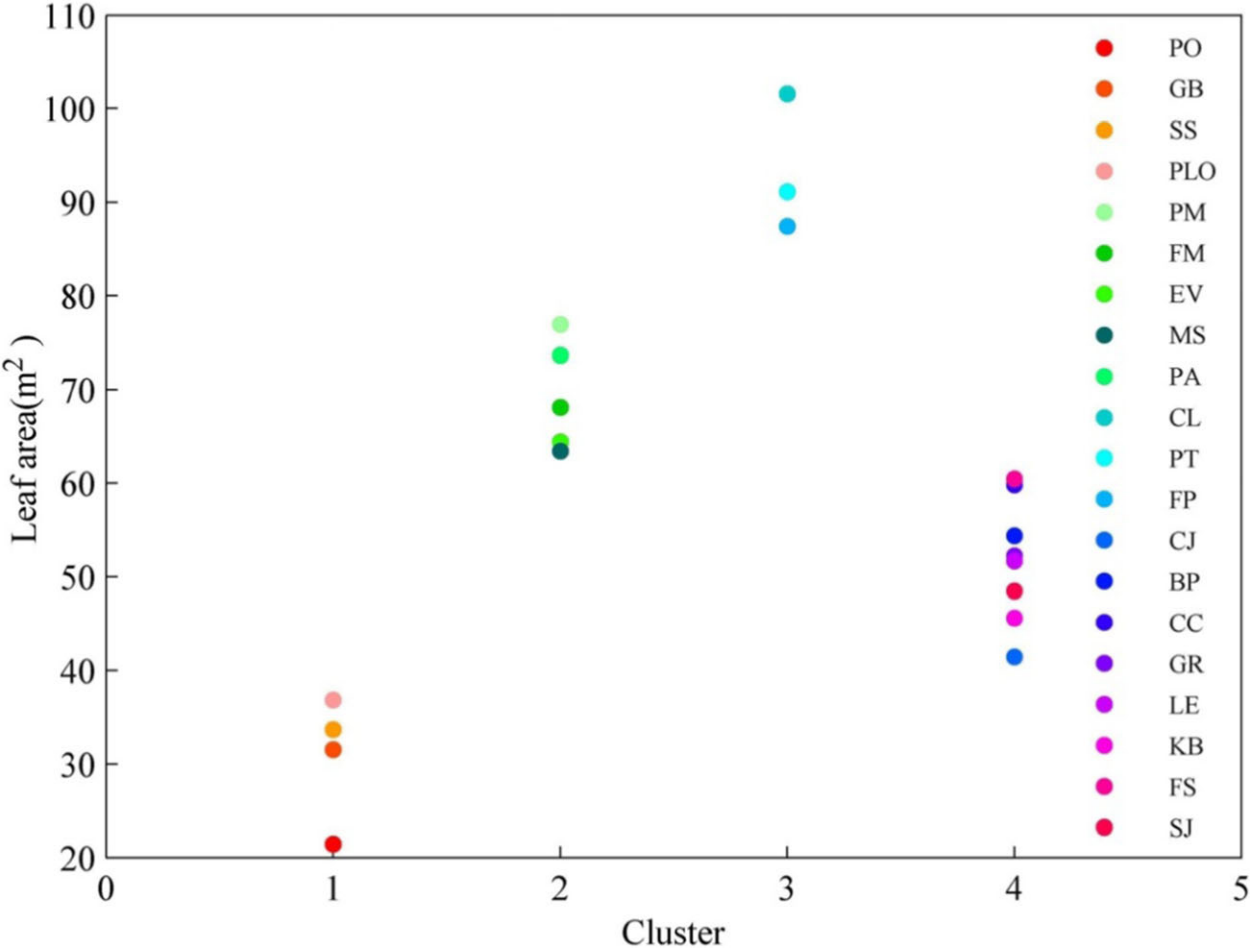
Cluster analysis of the total leaf areas per tree

Particle capture capability positively relate to the total leaf area per tree (Song et al. 2015). The difference in total leaf area per tree was examined by one**-**way ANOVA analysis and K-means clustering analysis. One**-**way ANOVA analysis showed that the total leaf area had significant difference among the 25 species (*P* < 0.01). Then, all the species were divided into four clusters (Fig. 4). The first cluster includes *P. orientalis*, *G. biloba*, *Symplocos setchuensis*, and *Platycladus orientalis*. Their leaf areas range from 21 to 37 m^2^. Species of the second cluster includes *P. massoniana*. *Ficus microcarpa*, *Erythrina variegate*, *Magnolia soulangeana*, and *Pinus armandii*, and have leaf areas ranging from 63 to 77 m^2^. The third cluster contains *Cunninghamia lanceolata*, *Populus tomentosa* and *Fraxinus pennsylvanica*, with leaf areas ranging from 87 to 102 m^2^. Others belong to the fourth cluster with a leaf area between 42 and 61 m^2^. We found that the total leaf area was superior for conifer (except *P. orientalis*) and lower for broadleaf species.

### Outcome of leaf morphology

Groove proportion ranges from 3 to 25 % and mostly between 10 and 20 %. There are about 50 % trees without leaf hair. Leaf hair of *B. papyrifera*, *Litsea elongata* in CQ, and *B. papyrifera*, *P. tomentosa* in BJ are 63.33 ± 11.60, 60.66 ± 7.14, 71.41 ± 9.28, 47.19 ± 9.41 mm^−2^, respectively, which is larger than others. *P. orientalis* in both cities and *F. microcarpa* in CQ have larger stomatal density and stomata size. *Cinnamomum camphora* in CQ and *M. soulangeana* in BJ have bigger stomata size.

**Fig. 5:**
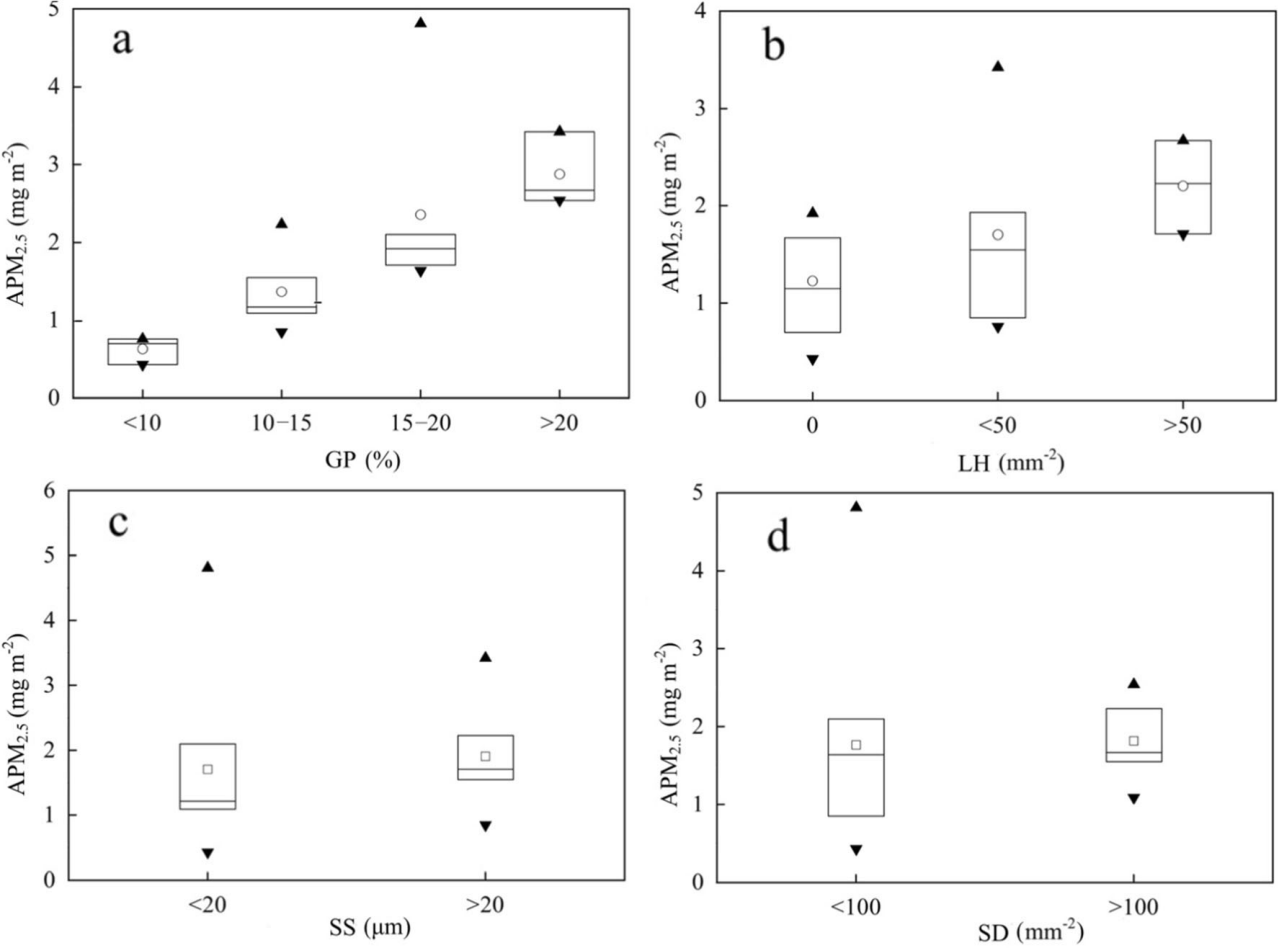
APM_2.5_ at different classified level (The *central rectangle* spans the first quartile to the third quartile and the *segment* inside the *rectangle* shows the median, while the *small squares* represent the average. The *triangle* above and below or overlapping the *dash* show the maximum and minimum value)

APM_2.5_ at different classified levels is shown in Fig. 5. In total, APM_2.5_ increase as the groove proportion and leaf hair rise. The APM_2.5_ is less than 1 mg m^−2^ when groove proportion is lower than 10 %. However, it sharply increases to 3 mg m^−2^ when groove proportion exceeds 20 %. The APM_2.5_ at different classified levels of leaf hair shows that (1) species without leaf hair seems to capture the lowest APM_2.5_ (1.20 mg m^−2^), (2) significant increase in APM_2.5_ (1.85 mg m^−2^) when leaf hair ranges from 0 to 50 mm^−2^, (3) largest APM_2.5_ exists when leaf hair exceed 50 mm^−2^. In spite of the little increase of APM_2.5_ with stomata size and stomatal density, APM_2.5_ concentrate at a high level when stomata size and stomatal density exceed 20 μm, 100 mm^−2^, respectively.

### Difference in capturing efficiency among the trees

After sedimentation, elution, and sample detection, captured PM_2.5_ per unit leaf area and per tree of each species were calculated (Table 2). A significant difference exists between species (*P* < 0.01). For the captured PM_2.5_ per unit leaf area, *C. lanceolate*, and *Grevillea robusta* in Chongqing had the highest PM_2.5_ capturing capability. However, *G. biloba*, *Cinnamomum japonicum*, and *M. soulangeana* shows the lowest PM_2.5_ capture capability. Captured PM_2.5_ of *G. biloba* is merely 10 % of *C. lanceolata*. Others range from 1.5 to 2.5 mg m^−2^. The results in Beijing indicate that the most efficient species are *B. papyrifera* and *Sophora japonica*. Besides, PM_2.5_ captured by conifer is lower than the actual, which can be explained by the fact that we ignored the wax layer and sticky secretions (Table 3).

**Table 2:** Statistics on leaf morphology

City	Type	Tree species	GP (%)	LH_a_(mm^−2^)	LH_b_(mm^−2^)	LH_t_(mm^−2^)	SD (mm^−2^)	SS(μm)
Chongqing	Broadleaf	***Platanus orientalis***	13.37 ± 1.87	0	2.33 ± 0.58	2.33 ± 0.58	37.21 ± 3.18	75.67 ± 7.59
		*Cinnamomum japonicum*	2.73 ± 0.76	0	0	0	8.84 ± 0.74	12.33 ± 1.34
		***Broussonetia papyrifera***	14.17 ± 2.08	20.33 ± 3.06	43.00 ± 8.54	63.33 ± 11.60	-	-
		*Cinnamomum camphora*	11.19 ± 2.81	0	0	0	15.64 ± 1.12	132.67 ± 15.42
		***Ginkgo biloba***	6.67 ± 1.51	0	0	0	17.24 ± 1.07	20.00 ± 3.98
		*Ficus virens*	12.47 ± 2.36	0	0	0	17.54 ± 0.98	30.33 ± 3.85
		*Ficus microcarpa*	13.81 ± 3.04	0	0	0	36.49 ± 2.89	138.67 ± 14.77
		*Erythrina variegata*	17.01 ± 3.41	0	0	0	20.07 ± 1.41	50.24 ± 3.47
		*Grevillea robusta*	23.66 ± 3.79	6.33 ± 1.52	12.67 ± 1.53	19.00 ± 3.05	22.54 ± 2.57	47.27 ± 3.18
		*Symplocos setchuensis*	13.77 ± 1.27	0	21.33 ± 6.51	21.33 ± 6.51	21.45 ± 2.87	112.84 ± 11.82
		*Litsea elongata*	21.05 ± 2.14	6.66 ± 0.58	44.00 ± 6.56	60.66 ± 7.14	17.34 ± 1.29	54.33 ± 9.54
		***Magnolia soulangeana***	3.86 ± 0.85	0	2.00 ± 1.00	2.00 ± 1.00	16.34 ± 1.61	98.67 ± 9.24
		*Koelreuteria bipinnat*	25.89 ± 2.10	7.00 ± 1.00	6.33 ± 1.15	13.33 ± 2.15	10.78 ± 0.75	192.47 ± 17.44
	Coniferous	*Cunninghamia lanceolata*	15.18 ± 1.87	0	0	0	14.21 ± 0.85	87.33 ± 6.87
		***Pinus massoniana***	16.18 ± 2.54	0	0	0	13.47 ± 1.98	32.33 ± 5.24
Beijing	Broadleaf	***Broussonetia papyrifera***	19.25 ± 1.59	25.21 ± 2.30	46.20 ± 6.98	71.41 ± 9.28	-	-
		***Ginkgo biloba***	9.23 ± 1.23	0	0	0	18.52 ± 1.07	20.00 ± 3.98
		***Platanus orientalis***	16.25 ± 2.05	0	2.58 ± 0.62	2.58 ± 0.62	38.36 ± 3.02	80.12 ± 6.26
		***Magnolia soulangeana***	4.03 ± 1.15	0	2.02 ± 0.96	2.02 ± 0.96	18.74 ± 1.61	100.36 ± 10.23
		*Populus tomentosa*	15.64 ± 1.83	7.86 ± 1.85	39.33 ± 7.56	47.19 ± 9.41	20.11 ± 2.07	81.53 ± 8.49
		*Fraxinus pennsylvanica*	15.98 ± 1.96	0	8.67 ± 1.82	8.67 ± 1.82	16.93 ± 1.76	67.87 ± 6.74
		*Sophora japonica*	12.11 ± 1.08	0	0	0	15.87 ± 1.69	76.84 ± 9.08
	Coniferous	*Platycladus orientalis*	13.28 ± 1.72	0	0	0	10.36 ± 1.85	41.17 ± 3.48
		*Pinus armandii* Franch.	16.57 ± 1.88	0	0	0	12.74 ± 2.14	35.17 ± 4.72
		***Pinus massoniana***	18.23 ± 2.20	0	0	0	14.47 ± 1.56	34.26 ± 6.59

Note: the bold are the common species in Chongqing and Beijing. GP represents groove proportion, LH_a_, LH_b_, and LH_t_ represents leaf hair in front, leaf hair on back, and total leaf hair, respectively

*SD* represents stomatal density, *SS* represents stomatal size

**Table 3:** Captured PM_2.5_ per unit leaf area and per tree and the total leaf area per tree of 25 species

City	Type	Tree species	Abbreviation	APM_2.5_ (mg m^−2^)	Total leaf area (m^2^)	Captured PM_2.5_ per tree (mg)
Chongqing	Broadleaf	***Platanus orientalis***	PO	0.81	21.43	17.3583
		*Cinnamomum japonicum*	CJ	0.72	41.43	29.8296
		***Broussonetia papyrifera***	BP	2.15	54.37	116.8955
		*Cinnamomum camphora*	CC	1.03	59.83	61.6249
		***Ginkgo biloba***	GB	0.45	31.53	14.1885
		*Ficus virens*	FS	1.25	60.45	75.5625
		*Ficus microcarpa*	FM	1.53	68.13	104.2389
		*Erythrina variegata*	EV	1.88	64.43	121.1284
		*Grevillea robusta*	GR	3.28	52.22	171.2816
		*Symplocos setchuensis*	SS	1.96	33.67	65.9932
		*Litsea elongata*	LE	2.63	51.67	135.8921
		***Magnolia soulangeana***	MS	0.72	63.42	45.6624
		*Koelreuteria bipinnat*	KB	2.52	45.62	114.9624
	Coniferous	*Cunninghamia lanceolata*	CL	4.72	102.56	484.0832
		***Pinus massoniana***	PM	2.02	76.96	155.4592
Beijing	Broadleaf	***Broussonetia papyrifera***	BP	2.35	54.37	127.7695
		***Ginkgo biloba***	GB	0.53	31.53	16.7109
		***Platanus orientalis***	PO	0.83	21.43	17.7869
		***Magnolia soulangeana***	MS	0.77	63.42	48.8334
		*Populus tomentosa*	PT	1.87	91.14	170.4318
		*Fraxinus pennsylvanica*	FP	1.07	87.47	93.5929
		*Sophora japonica*	SJ	2.12	48.47	102.7564
	Coniferous	*Platycladus orientalis*	PLO	1.24	36.82	45.6568
		*Pinus armandii*	PA	1.7	73.65	125.205
		***Pinus massoniana***	PM	2	76.96	153.92

Note: The bold are the common species in Chongqing and Beijing. APM_2.5_ represents captured PM_2.5_ per unit leaf area

For the captured PM_2.5_ per tree, *C. lanceolata* captures the largest PM_2.5_. *G. robusta*, *P. massoniana*, and *Litsea elongate* are efficient in Chongqing. *B. papyrifera* is the most inefficient species due to the lower leaf area. Others range from 14.00 to 123.00 mg per tree. In Beijing, *P. tomentosa* shows the highest PM_2.5_ capture capability, while *P. massoniana*, *B. papyrifera*, and *P. armandii* are the intermediate ones among the 25 tree species. *G. biloba*, *P. orientalis*, and *M. soulangeana* belong to the lowest species. *B. papyrifera* and *P. massoniana* have comparatively high PM_2.5_ capture capability. However, the same species in Beijing capture more PM_2.5_ than those in Chongqing.

All the leaf characteristics data were normalized using SPSS (vision 19.0) to obtain dimensionless data including captured PM_2.5_ per leaf area (ZM), groove proportion (ZGP), leaf hairs (ZLH), stomata size (ZSS), and stomatal density (ZSD). Results found that there were significantly positive correlation between ZM and ZGP, ZSS (*p* < 0.01). A significant correlation between ZM, ZLH, and ZSD were not found (*p* > 0.05).

## Discussion

The PM_2.5_ capture capability of 25 species in Beijing and Chongqing were tested by a chamber device. We obtained PM_2.5_ capture capability per unit leaf area and per tree, which can be helpful for selecting proper vegetation in urban settings. We tested the same species in Chongqing and Beijing at the same environment (same pollution level, temperature, and humidity). Surprisingly, there was a difference between the two sites. It can be seen that the APM_2.5_ in Beijing were larger, possibly due to the rich leaf morphology.

Leaf features of coniferous species can cause high air turbulence inside the tree crowns, leading to an increase in the interception capacity of contaminants (Bunzl et al. 1989). In this study, conifers did not show a significant advantage to capture APM_2.5_ compared with broadleaf species. However, PM_2.5_ accumulation capacity of conifers was superior to most broadleaf species for larger leaf areas per tree.

**Fig. 6:**
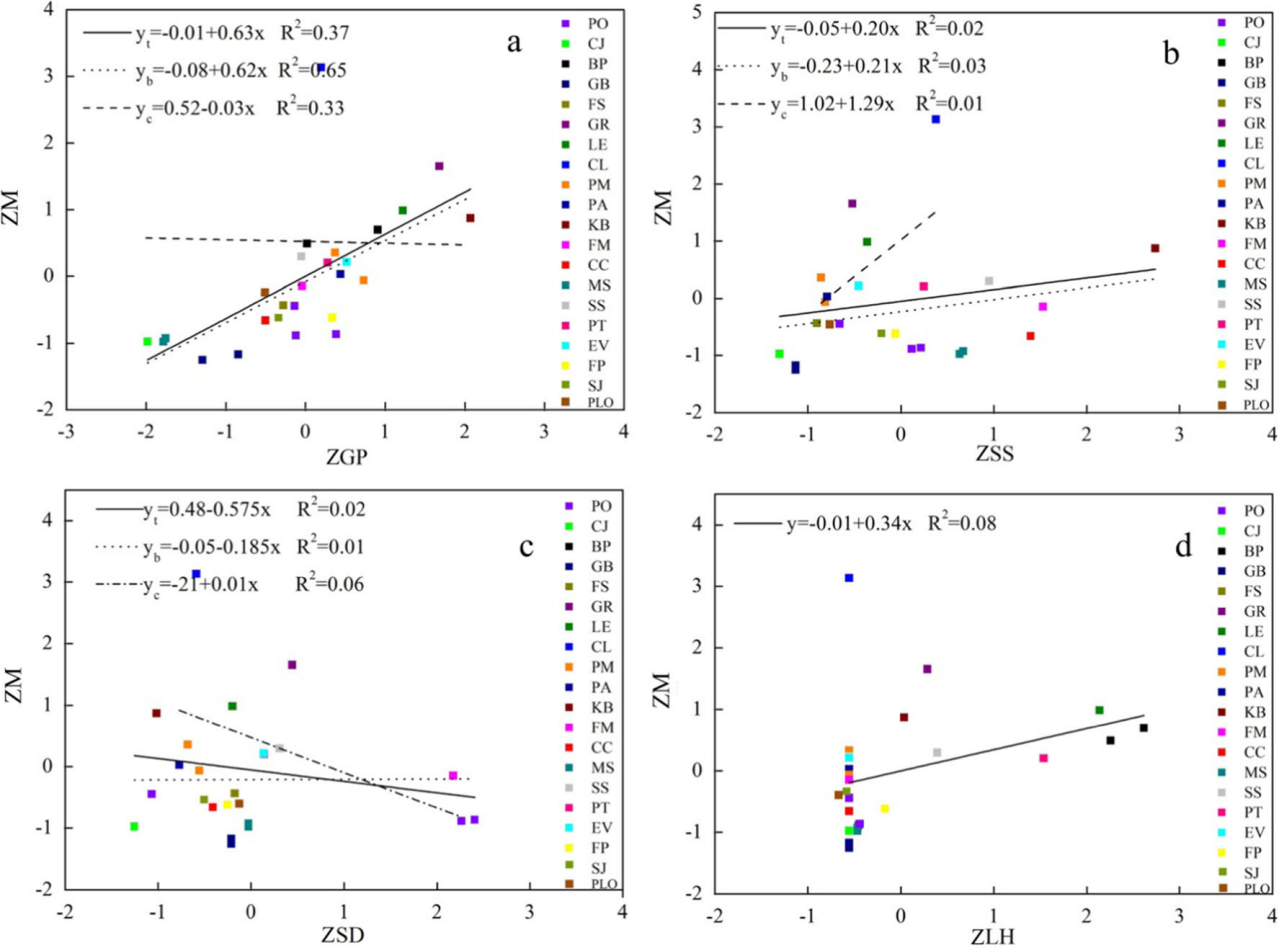
Correlation analysis of normalized leaf morphology and captured PM_2.5_ per unit leaf area (y_b_ represents the species in Beijing; y_c_ represents the species in Chongqing; y_t_ represents the species in both Chongqing and Beijing. Each *colored small square* represents a tree species)

Leaf morphology appears to be a dominant factor in particle deposition (Mitchell et al. 2010). In previous studies, leaf morphology was qualitatively analyzed, while few quantified results were made (Chai et al. 2002). More detailed classifications and quantification of leaf morphology need to be further refined. In our study, groove proportions were quantified to evaluate the roughness. Meanwhile, leaf hair and stomatal density were quantified by counting them in a fixed leaf area. In addition, some studies revealed that particulate matter can get into leaves through the stomata, where fine particulates often crowded (Song et al. 2015; Lehndorff et al. 2006). Therefore, stomata size was also quantified. Our main results reveal that grooves are the main parts of a blade that capture PM_2.5_. A strong correlation between the PM_2.5_ accumulation and groove proportion proves that leaf surface roughness is a facilitator for PM_2.5_ capture (Fig. 6a). In addition, stomata size is an important influence factor for PM_2.5_ capture capability.

In this research, NaCl was used as a PM_2.5_ source, thus the mass of NaCl is lower than the natural PM_2.5_ source. The mass of captured PM_2.5_ per tree in our study was lower than the results in Song et al. (2015). Overall, the method used in this study is suitable for comparing the difference between species qualitatively merely.

### Leaf characteristic and leaf morphology

Though all sampled species can capture PM_2.5_ and leaf surfaces have a considerable capacity (Wang et al. 2015), the amount differ significantly depending on leaf morphology. *B. papyrifera* is the most hairy for both lower and upper sides of the leaf and has the high groove proportion. The stomata size and stomatal density of *B. papyrifera* are not counted because leaf hair covered the stoma on leaf surface. *Koelreuteria bipinnat* has the highest groove proportion and stomata size, while the leaf hair and stomatal density of *K. bipinnat* are low. All of them do not show the highest PM_2.5_ capture capability. *G. robusta* is the most efficient broadleaf species and *C. lanceolate* is the most efficient conifer with high groove proportion and low stomata size. Species with high groove proportion and low stomata size is most effective at capturing PM_2.5_. Earlier studies also reported that mounts of PM_2.5_ captured on rough tree leaves with low stomatal density were high (Hwang et al. 2011; Räsänen et al. 2013).

Nguyen et al. (2015) found that trees with leaf hairs have high PM_2.5_ capture capability. Species with densely haired leaves were most effective at capturing PM (Dzierżanowski et al., 2011; Weber et al. 2014). However, we found no significant correlation between PM_2.5_ capture capability and leaf hair (Fig. 6d). This may attribute to differed methodological approaches and limited tree species in this research. A large number of tree species need to be studied in the future.

**Fig. 7:**
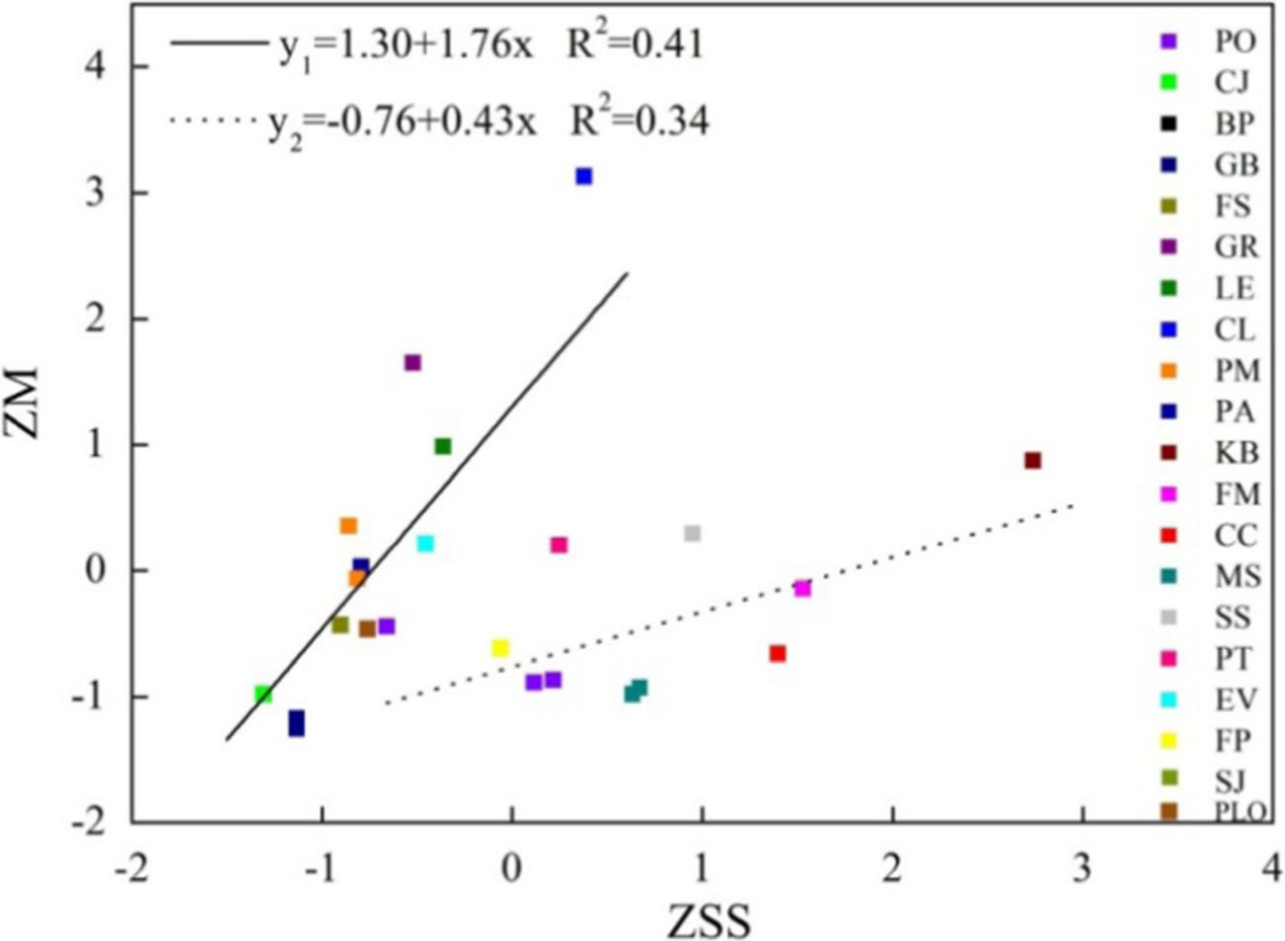
Correlation analysis of normalized stomata size and captured PM_2.5_ per unit leaf area (y_1:_ CJ, BP, GB, FS, GR, LE, CL, PM, PA, EV, SJ, PLO; y_2:_ PO, MS, CC, FM, KB, SS, PT, FP)

The role of stoma activity in particle deposition is ambiguous. On one hand, transpiration of water through stomata cools the surface which is conducive to attracting PM_2.5_; on the other hand, transpired water repels PM_2.5_ due to diffusiophoresis (Hinds 1999). No statistically significant correlations are found between PM_2.5_ capture capability and stomata size (Fig. 6b), stomatal density (Fig. 6c) in Chongqing and Beijing. For the stomata size, significant correlation with PM_2.5_ accumulation exists when tree species are classified into two groups according to stoma size:(1) The bigger one: PO, MS, CC, FM, KB, SS, PT, FP, which average stomata size are 111.19 μm. (2) The smaller one: CJ, BP, GB, FS, GR, LE, CL, PM, PA, EV, SJ, PLO, which average stomata size are 44.41 μm (Fig. 7). It can be seen that the correlation of the smaller one is higher than the bigger one, which can be explained by the restrain effect due to small stomata size. When the stomata size grow, the restrain effect become smaller.

### Difference between coniferous and broadleaf species

Conifers shows the highest particle capture efficiency of tested tree species, which coincides with previous studies. Among the conifers, pines captured significantly more PM_2.5_ than cypresses (Beckett et al., 2000a, b). In this study, *C. lanceolata* is the most effective species in PM_2.5_ accumulation. Nevertheless, *P. orientalis* belongs to cypresses and has the least efficiency of PM_2.5_ accumulation, which coincides with the results of Song et al. (2015). It may be due to that pine trees deposited more PM_2.5_ than cypress ones.

**Fig. 8:**
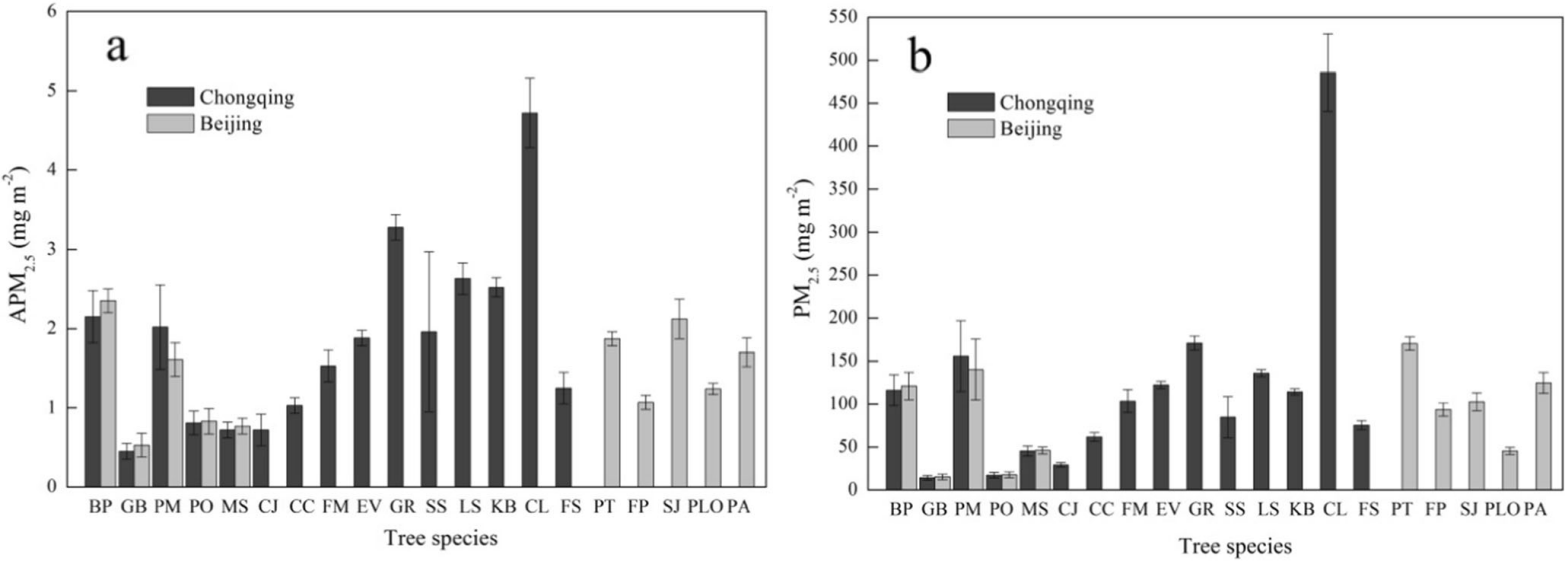
Captured PM_2.5_ of different species in Beijing and Chongqing

More complex structure of the foliage of the conifers explained their greater effectiveness at capturing particles (Beckett et al., 2000a, b). However, in this study, conifers did not show a significant advantage to capture APM_2.5_ comparing with broadleaf species (Fig. 8a), which may attribute to that the structure of the conifer crowns were not considered. The total leaf area per tree of the conifer is higher than broadleaf species. Therefore, PM_2.5_ accumulation capacity per tree of conifers are superior to most of broadleaf species (Fig. 8b).

In addition, some trees are better able to survive in smoky and polluted conditions due to differences in physiological mechanisms of varied species. All in all, the best choices for pollution-control plantings are coniferous and broadleaved species with rough leaf surfaces and high adaptability (Beckett et al. 1998, 2000a, b; Silli et al. 2015).

### Difference between two sites

A number of studies have demonstrated the effects of pollution on tree leaves. Deposition of PM was responsible for the change on leaf surface morphology (Gupta et al. 2015). Furthermore, it was found that the effects of PM_2.5_ on leaves relate to their acidity, salinity, and trace metal content properties (Grantz et al. 2003). Leaf density and thickness are altered when exposed to pollution environment and higher levels of NO_X_ (Jochner et al. 2015). Pääkkönen et al. (1997) found that higher stomatal density and thicker leaves result in a greater tolerance to pollution. In addition, it was likely that PM_2.5_ might have an indirect effect via altering soil chemistry, which is also believed to be the major effect of PM on trees (Grantz et al. 2003). Trees strengthened the characteristics of their leaf structures under polluted conditions, which are regarded as adaptive and compensative to the adverse effects of air pollution (Chaturvedi et al. 2013). Studies also showed that trees develop different morphologies under polluted conditions (Karenlampi 1986; Veselkin 2004).

In addition, the chemical composition and wax structure may also be different in Beijing and Chongqing, which are significant for PM_2.5_ capture (Burkhardt 2010). Therefore, it is worthy of further study regarding the effect of PM_2.5_ pollutions on leaf morphology, including chemical composition, wax structure, groove proportion, leaf hair, stomatal density, and stomata size.

## Summary and conclusion

This study revealed that broadleaf species with rich leaf morphology, namely, leaf groove, leaf hair and stomata, can capture more PM_2.5_ per leaf area than coniferous. However, coniferous captured larger PM_2.5_ per tree due to their large leaf area per tree. Among coniferous, *C. lanceolata*, *P. orientalis*, *P. armandii* were most efficient in capturing PM_2.5_. *G. robusta*, *Erythrina variegata*, *K. bipinnata*, *P. tomentosa*, *F. pennsylvanica* showed relatively high PM_2.5_ accumulations. A difference exists between the trees in Beijing and Chongqing due to the environment and leaf characteristic difference. Groove proportion and stoma size positively relate to PM_2.5_ accumulations, while there is no significant correlation between PM_2.5_ capture and stomatal density and leaf hair. Efficiency of PM_2.5_ capture capability in this study was examined by gas chamber, which is worthy of further analysis in field conditions. Thus, climate conditions, urban planning and management, and advantage tree species should be considered when greening. Urban greening trees are diverse and the structures are complicated. Complex ecological environment may lead to different physiological characteristics. Hence, it is valuable to examine the PM_2.5_ accumulation capability in specific ecological environment and different growth stages of trees. In addition, physiological indicators such as leaf photosynthetic rate, transpiration rate and stomatal conductance should be considered.
